# Shifts in predator behaviour following climate induced disturbance on coral reefs

**DOI:** 10.1098/rspb.2022.1431

**Published:** 2022-12-21

**Authors:** Randi D. Rotjan, Nicholas E. Ray, Ingrid Cole, Kurt G. Castro, Brian R. C. Kennedy, Tina Barbasch, Kathryn C. Lesneski, Karina Scavo Lord, Anjali Bhardwaj, Madeleine Edens, Ioanna Karageorge, Caitlynn Klawon, Hallie Kruh-Needleman, Gretchen McCarthy, Raziel Perez, Christopher Roberts, Isabela F. Trumble, Aryanna Volk, Javon Torres, Joshua Morey

**Affiliations:** ^1^ Department of Biology, Boston University, 5 Cummington Mall, Boston, MA 02215, USA; ^2^ Boston University Marine Program, Boston University, 5 Cummington Mall, Boston, MA 02215, USA; ^3^ Department of Ecology and Evolutionary Biology, Cornell University, Ithaca, NY 14853, USA; ^4^ University of Belize, Belize City, Belize

**Keywords:** coral bleaching, corallivory, parrotfish, herbivory, global change

## Abstract

Coral reefs are increasingly ecologically destabilized across the globe due to climate change. Behavioural plasticity in corallivore behaviour and short-term trophic ecology in response to bleaching events may influence the extent and severity of coral bleaching and subsequent recovery potential, yet our understanding of these interactions *in situ* remains unclear. Here, we investigated interactions between corallivory and coral bleaching during a severe high thermal event (10.3-degree heating weeks) in Belize. We found that parrotfish changed their grazing behaviour in response to bleaching by selectively avoiding bleached *Orbicella* spp. colonies regardless of bleaching severity or coral size. For bleached corals, we hypothesize that this short-term respite from corallivory may temporarily buffer coral energy budgets by not redirecting energetic resources to wound healing, and may therefore enable compensatory nutrient acquisition. However, colonies that had previously been heavily grazed were also more susceptible to bleaching, which is likely to increase mortality risk. Thus, short-term respite from corallivory during bleaching may not be sufficient to functionally rescue corals during prolonged bleaching. Such pairwise interactions and behavioural shifts in response to disturbance may appear small scale and short term, but have the potential to fundamentally alter ecological outcomes, especially in already-degraded ecosystems that are vulnerable and sensitive to change.

## 1. Introduction

Evolutionary interactions on coral reefs rely on relative ecological predictability due to the relatively constant environmental conditions in the tropics, with regular annual and lunar growth cycles well-documented in corals [1]. Although the relatively stable conditions on reefs have been previously disturbed over geological time [2,3], reefs are currently suffering from the consequences of anthropogenically driven climate change on a global scale [4,5]. In particular, climate change causes myriad and intensified disturbances on coral reefs globally, leading to a well-documented series of consequences ranging from bleaching stress (dysbiosis) to subsequent mortality of both the corals and other reef inhabitants due either to increased temperature or related factors [6–8] that erode ecological resilience [9,10]. There have been many recent advances in understanding the proximate and ultimate causes of bleaching [8,9], the role of symbiosis in the dynamics of bleaching [10–12] and the consequences of bleaching [7,8], yet the immediate, real-time ecological dynamics of *in situ* bleaching on the behaviour of coral-dependent reef communities, specifically the impact on short-term trophic ecology and corallivore behaviour, remains unclear.

Coral bleaching is a relatively short-lived phenomenon, typically lasting days to weeks prior to either recovery or subsequent mortality [13,14] and it is thus assumed that most reef community dynamics remain intact during the short period of actual bleaching. However, coral bleaching and resulting coral host stress likely have real-time influences on corallivore behavioural ecology, nutritional ecology and trophic transfer on a reef. Scleractinian corals are key primary producers on reefs [15], mainly resulting from their classic symbiosis with photosynthetic dinoflagellates (Symbiodiniaceae). Global net primary production of reefs can be as high as 20 Tg C yr^−1^ [16]. This productivity is transferred to the reef environment in different ways, but one of the main mechanisms of trophic transfer from corals to the food web is via corallivory, the consumption of live coral [17,18]. Because corallivores can exhibit behavioural plasticity in response to bleaching [17–19], they have the potential to profoundly alter reef community dynamics in real time. Further, corallivore behaviour may have the potential to influence the fate of coral resilience; for example, increased grazing may decrease coral resilience. Despite their potential ecological importance, the interaction between bleaching and real-time corallivory has not been well-studied [19].

To date, the limited number of studies on real-time bleaching–corallivory interactions have all been conducted with obligate corallivores, most have been conducted under laboratory (not environmental) bleaching conditions, and in total have shown mixed results. For example, grazing by the tube-lip wrasse, *Labrichthys unilineatus*, initially increased as corals began to bleach, but then ceased when colonies were fully bleached [20]. By contrast, the eastern triangle butterflyfish, *Chaetodon baronessa* [20] and the blueblotch butterflyfish, *Chaetodon plebius* [21] consistently preferred healthy colonies over bleached colonies, even during early onset bleaching. *Chaetodon ornatissimus*, *Chaetodon pelewensis* and *Chaetodon reticulatus* also prefer healthy over bleached colonies, but the strength of this selectivity appears to be depth-dependent [22]. The oval butterflyfish (*Chaetodon lunulatus*) does not appear to show grazing discrimination based on bleached status [21]. Thus, existing information on real-time impacts of bleaching on feeding behaviour and resulting trophic transfer are sparse, may differ on a taxa-specific basis, and are limited to studies on only a few obligate corallivores.

All Caribbean corallivorous fishes are facultative [17,18]. Of these, the most prevalent are the parrotfishes (Scaridae) and butterflyfishes (Chaetodontidae), both of which comprise multiple species that are highly mobile and graze live corals amidst other food items such as algae, small invertebrates and detritus [18,23–25]. Observations of butterflyfish feeding in real time during bleaching would be the only way to assess behavioural responses [22], because butterflyfishes do not leave a visible grazing scar. Parrotfishes (namely *Sparisoma* and *Scarus* spp.) leave scars that are both visible (white skeleton exposed) and textural (polyps removed with resulting skeletal abrasion). Further, grazing scar age is relatively simple to assess, as fresh grazing scars have no turf algal growth. As such, scars represent clear evidence of grazing even in the absence of direct behavioural observations, and can serve as an indicator of recent fish grazing behaviour.

During coral bleaching events, *Orbicella* spp. corals are often highly susceptible and among the most vulnerable in the Caribbean [26,27], though their vulnerability changes based on host condition and biomass [28]. Their flexible association with Symbiodiniaceae species [29–31] often leaves colonies at least partially bleached when waters exceed 8-degree heating weeks (DHW;°C-weeks) on reefs [32], though different populations have different thermal optima and bleaching thresholds [33]. *Orbicella* spp. corals are large, hermatypic reef builders that are major contributors to overall reef matrix and structure, and have provided important habitat on Caribbean reefs from shallow backreef environments to the mesophotic since at least the Late Pleistocene [34]. *Orbicella* spp. are also among preferred prey of major Caribbean corallivores [35,36]. Chronic corallivory by parrotfishes hinders the recovery of *Symbiodininiaceae* populations in *Orbicella* spp. following bleaching and changes the community composition of *Symbiodiniaceae* within bleached and grazed colonies [37]. The influence of parrotfish grazing on bleaching intensity, or the influence of bleaching on real-time parrotfish corallivore grazing behaviour is less clear.

In this study, we investigated the interaction between corallivory and bleaching on massive *Orbicella* spp. colonies in Belize. Beginning in August 2019, Turneffe Atoll experienced a severe bleaching event with a maximum of 10.3 DHW, a measure of cumulative heat stress over a 12-week period. We measured the per cent of *Orbicella* spp. colonies that were bleached in 2019 and over 2300 grazing scars on bleached and unbleached *Orbicella* spp. corals at three sites in Belize to determine parrotfish grazing preferences during active bleaching. We also investigated whether heavily grazed colonies experienced higher bleaching intensity, and examined whether large coral colonies exhibited more resilience, or vulnerability to bleaching and/or grazing. Finally, we compared the molar ratio of carbon (C) to nitrogen (N; C:N), %C and %N and δ^13^C and δ^15^N of corals in response to grazing and/or bleaching. This study represents the first to examine the ecological real-time trophic consequences of bleaching on parrotfish corallivory on tropical reefs.

## 2. Methods

**Figure 1.**
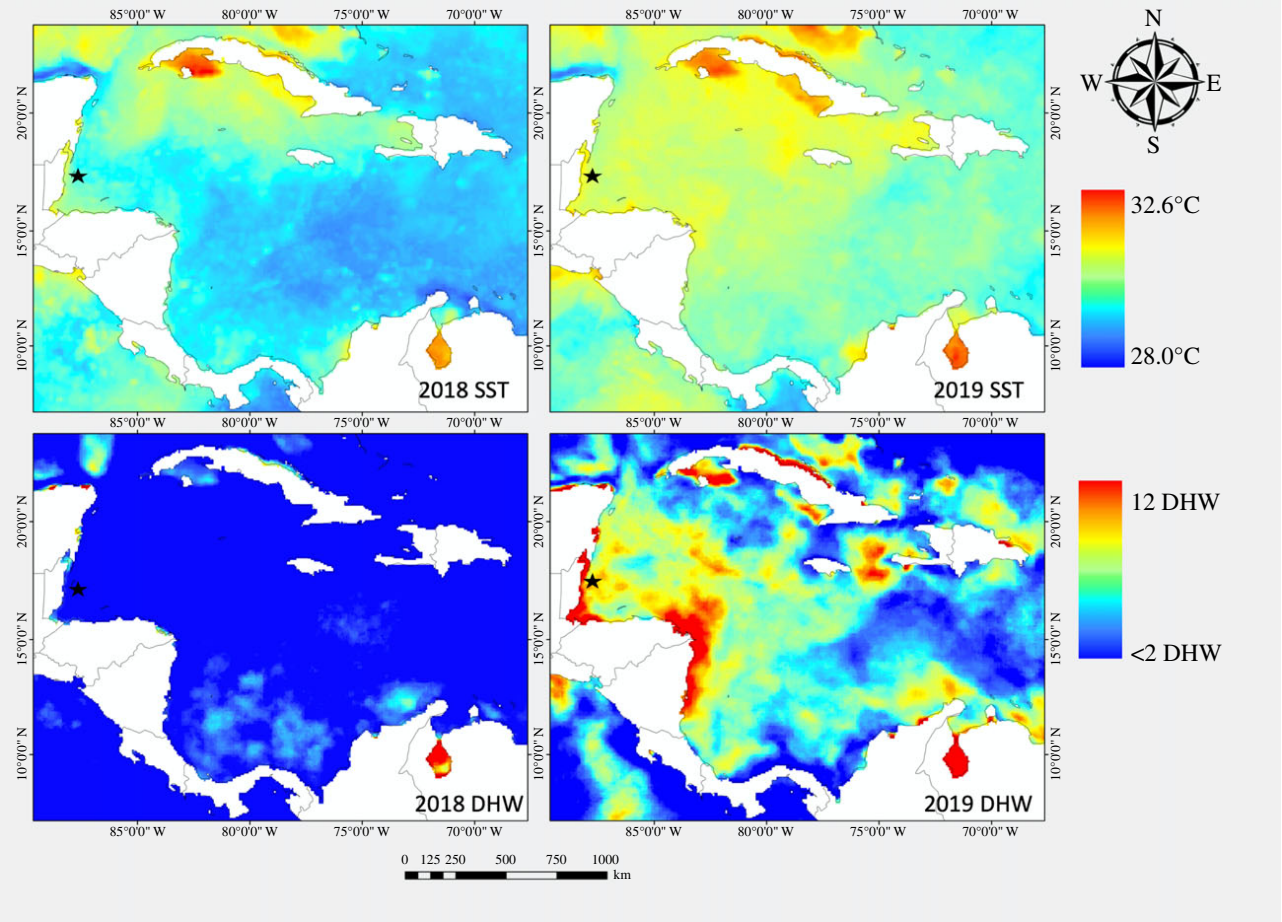
Maximum sea surface temperature (SST) and degree heating weeks (DHW) in the Caribbean in 2018 and 2019. Data are shown for all of Belize, including the Meso-American Barrier Reef and outer Atolls. Turneffe Atoll is the largest of the three. (Online version in colour.)

The data used in this study were collected as part of a Boston University ‘Coral Reef Dynamics' course (BI 539) taught on Turneffe Atoll, Belize (electronic supplementary material, figure S1) by R. Rotjan and T. Barbasch in 2018 and by R. Rotjan and N. Ray in 2019. Boston University graduate and undergraduate students collected these data as part of the course, with careful oversight and assistance from the teaching staff. Courses were run in December 2018 and December 2019; bleaching occurred in 2019 (detailed below and in figure 1).

### (a) Study site and approach

**Figure 2.**
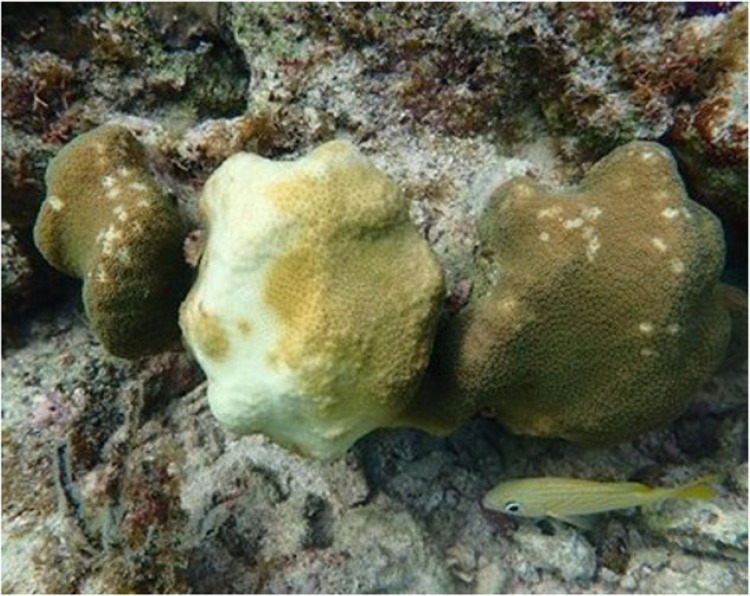
Example of a partially bleached *Orbicella* spp. colony with corallivory clearly visible on the unbleached sections. (Online version in colour.)

Four reefs were investigated during the study (electronic supplementary material, figure S1). At three of these reefs, we surveyed bleached versus non-bleached *Orbicella* spp. corals to examine the number of grazing scars, colony area and bleaching extent in 2019. These sites were selected with the criteria that (i) there were a minimum of 50 *Orbicella* spp. colonies within snorkel depths (less than 3 m) and (ii) there was evidence of recent parrotfish corallivory (so that we could examine grazing/bleaching interactions; figure 2). These sites include Major's Reef: a patch reef (*n* = 50 corals), Wonderland: a shallow fore reef (*n* = 70 corals) and South of the Border: a patch reef (*n* = 71 corals; electronic supplementary material, figure S1). At each of these three reefs, we surveyed all *Orbicella* spp. heads that we could find, noting that—especially for *O. annularis*—there were multiple subheads that might be bleached and some that might be unbleached on the same colony (figure 2). In this way, the number of coral sections sometimes exceeded the number of coral colonies. At each coral head a picture was taken with a Coral Watch Health Chart [38] from which the per cent area and degree of bleaching was calculated from a scale of 1 (severely bleached) to 6 (not bleached). Per cent bleaching was also estimated in the field (with two to three observers each independently estimating bleaching severity and then comparing and averaging with each other); this method was used throughout. Dimensions (length, width, height) of each coral head were measured to calculate coral area as an indicator of size. Many of the colonies we surveyed had distinct areas of bleaching. We then counted the number of bite scars, which were clearly visible on coral heads and were easily counted *in situ* (figure 2). When counting bite scars, we assigned scar counts to bleached and unbleached areas on each colony separately. Over 2300 individual grazing scars were assessed for this analysis. After surveying each coral head we placed a biodegradable marker made from a coral rock with a palm frond tied around it next to the coral head to avoid resurveying it.

The fourth site, Calabash Apron, is a long-term monitoring site, and was used to compare grazing data collected from 8 to 17 December 2018 with bleaching data collected from 7 to 16 December 2019. Detailed methods for each question investigated are described in §2*a*–*g* below.

### (b) Description of the 2019 bleaching event

We used the NOAA Coral Reef Watch CoralTemp database to determine the DHW and maximum sea surface temperature (SST) in 2018 and 2019 to characterize the 2019 bleaching event (figure 1). Maps were created with ESRI Arc GIS v.10.6. We calculated the percentage of *Orbicella* spp. colonies at the Calabash Apron site that were bleached in both 2018 and 2019 using three-dimensional photomosaics compiled in Agisoft v.1.4.4. This method allowed us to fully census all colonies within a known area (747.1 m^2^ in 2018 and 1201.7 m^2^ in 2019) to determine bleaching extent. At the Calabash Apron site, we also used 84 tagged *Orbicella* spp. colonies to assess changes in grazing scars between years.

### (c) Do parrotfish selectively avoid grazing on bleached *Orbicella* spp. colonies?

We used data collected from Major's Reef, Wonderland and South of the Border to test whether unbleached coral sections were grazed more frequently and had more bites than unbleached coral sections. We excluded Calabash Apron as there was no evidence of recent corallivory in December 2019. To statistically test whether unbleached coral sections were grazed more frequently and had more bites than unbleached coral sections, we used a hurdle model approach [39] via the *pscl* package in R Statistical Software [40]. In this study and for our analyses, we considered ‘bleached’ sections of coral to be scores 1–3 on the coral watch card, and ‘unbleached’ as 4–6. We considered the results of this and all other statistical tests reported in our study to be significant when *p* ≤ 0.05. We used the *ggplot2* package [41] or Graphpad Prism (v.8.4.2) to make all figures.

### (d) Does grazing density vary with bleaching severity?

To compare the number of bites on grazed sections of varying bleaching, we constructed a general linear model and compared the average number of bites in sections of different bleaching scores recorded at Wonderland, Majors Reef and South of the Border using pairwise comparisons via a least square means test.

### (e) Does coral size influence bleaching or grazing susceptibility?

To test for potential relationships between coral size and susceptibility to bleaching (i.e. per cent of the colony that was bleached) and grazing (i.e. number of grazing scars), we used a linear regression approach. At each of the three reef sites with recent *in situ* parrotfish grazing, we used the length, width and height of each colony to estimate a surface area, using the formula for an ellipsoid (equation (2.1)):

2.1 $$\mathrm{Surface}\;\mathrm{area}=4\pi {\left(\frac{lw^{p}+\;lh^{p}+\;wh^{p}}{3}\right)}^{1/p}$$

where *l*, *w* and *h* are the colony length, width and height, respectively, and *p* is a constant equal to 1.6075. We then divided this value by two, to account for just the exposed area of the colony (approximately half a spheroid) and calculated linear regressions using surface area to predict the per cent of the colony that was bleached, or the number of grazing scars.

### (f) Do heavily grazed colonies suffer higher bleaching severity?

To examine the relationship between grazing intensity and bleaching severity, we examined bleached *Orbicella* spp. coral colonies from Calabash Apron. In total, 84 colonies were tagged and were examined for grazing scars *in situ* in 2018 and were found and re-surveyed in 2019. We carefully examined each colony to count the number of bite scars each year, but in December 2019, when we attempted to quantify the number of bite scars, we found none, despite observing numerous parrotfish in the area. We also examined these 84 tagged colonies for bleaching, and the relative per cent bleaching was estimated *in situ* using a colour chart, as above. The best model to describe the relationship between grazing scars in 2018 and per cent of the colony bleached in 2019 required a log transformation, so we added a value of 1 to all per cent scores in order to make this transformation (as zero values cannot be log transformed). To corroborate *in situ* bleaching observations across years, we cross-checked our field data with top-down photomosaic surveys of 411 colonies in 2018, and 684 colonies in 2019. Photos were taken with Olympus Tough TG-5 underwater cameras by taking overlapping photographs of a large (roughly 30 × 30 sq m) plot on snorkel. Making sure that images overlapped by at least 1/3 view to enable high-quality post-processing, 3914 photographs were taken in 2018 and 4092 photographs were taken in 2019. Photomosaics were scaled using three 0.5 m PVC pipes as size references that were placed on the reef during photo collection. Photomosaics were compiled in Agisoft Photoscan (now known as Metashape) v.1.4.4 using the manufacturer recommended procedure. The top-down mosaics were used to determine the spatial extent of the study area and to corroborate *in situ* data.

### (g) Is there a difference in coral tissue quality between bleached and unbleached colonies?

Coral tissue can be useful as an indicator of coral trophic quality, as well as indicate the source of nutrients to the coral. We collected coral samples from bleached colonies (*N* = 6) and unbleached colonies (*N* = 9) for analysis of C and N content and isotopic ratio from Majors Reef, Calabash Apron and Wonderland using a hammer and chisel. Samples were frozen and transported to Boston University, where tissue was separated from the skeleton using a WaterPik. The slurry created during this process was collected and centrifuged. We collected the pellet created during centrifugation and dried it with a food dehydrator until achieving a constant weight [42]. Approximately 1.5 mg of the dry tissue from each pellet sample was loaded into tin capsules (Costech Analytical Technologies, Inc.) for analysis in the Boston University Stable Isotope Lab using a Eurovector CN analyser connected to a continuous flow GV Instruments Isoprime Isotope Ratio Mass Spectrometer. External precision standards were 0.2‰ for δ^15^N and δ^13^C. For 13CV-PDB the gas is calibrated against 64 NBS 20 (Solnhofen Limestone). For ^15^N air the gas is calibrated against atmospheric N_2_ and IAEA standards N-1, N-2 and N-3 (all are ammonium sulfate standards). Technical replicates were conducted on all samples to ensure precision. All were within the min./max. range of other individuals in the same treatment. All data are reported in per mille (‰) values. We used the mean value of these technical replicates to compare %C, %N, δ^15^N and δ^13^C between bleached and unbleached coral using generalized linear models with bleaching status as a fixed effect and the reef they were collected from as a random effect, followed by least square means tests. For one unbleached sample, the instrument did not record the C peak, and this sample was excluded from our analysis.

## 3. Results

### (a) Description of the 2019 bleaching event

In 2019, the coral reefs around Turneffe Atoll experienced a severe bleaching event that began the second week in August. At the height of the bleaching event, the reefs experienced a maximum of 10.3 DHW on 4 November 2019 and reached a maximum of 30.1°C SST on 25 September 2019. For the purposes of our study, we plotted DHW and SST from the beginning of the event through the last day of our survey in 2019 (16 December 2019), compared to the same time period in 2018 (figure 1). From compiled photomosaic images from the Calabash Apron site, we calculated an *Orbicella* spp. bleaching rate of 0% in 2018 (*n* = 411 colonies over 747.1 m^2^) and 87.4% in 2019 (*n* = 684 colonies over 1201.7 m^2^).

### (b) Parrotfish selectively avoid grazing on bleached *Orbicella* spp. colonies

**Figure 3.**
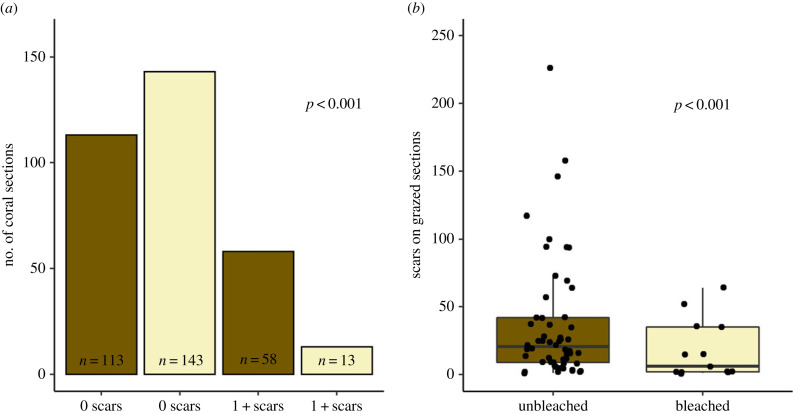
(*a*) Number of unbleached (brown/dark bars) and bleached (white/light bars) coral sections with parrotfish bite marks and (*b*) the number of bite marks on grazed coral sections in 2019. *p*-Values indicate the results of a hurdle model comparing the likelihood of a coral section having bite marks based on bleaching condition (*a*) and the total number of bites on grazed sections (*b*). Each point on the box plot represents the number of bites on a single coral section. (Online version in colour.)

We measured bleaching and parrotfish grazing on 178 *Orbicella* spp. colonies at Majors Reef, Wonderland and South of the Border in 2019 with active bleaching (table 1). The average per cent area of coral colony bleaching across all sites was 43.09% ± 2.47 (standard error; from here on, all values are reported as mean ± s.e.). Not only were unbleached coral sections on these colonies at least four times more likely to have bite scars than bleached coral sections (*p* = 0.001; figure 3*a*), they also had twice as many scars (36.46 ± 5.96 bite marks section^−1^) compared to bleached sections that exhibited at least some grazing (18.00 ± 6.00 bite marks section^−1^; *p* < 0.001; figure 3*b*).

**Table 1.** Number of samples collected at each site. The values in the % bleached column represent the mean ± s.e. of the amount of the colony that was bleached. In some situations, there were more coral sections than colonies due to the colony shape, and distribution of bleached and unbleached sections.

site	colonies surveyed	unbleached sections	bleached sections	% area of coral bleached
Major's Reef	49	50	47	47.22 ± 4.20
South of the Border	57	61	47	32.29 ± 3.81
Wonderland	70	66	58	49.64 ± 4.41

### (c) Diminished grazing intensity does not correspond with bleaching severity

**Figure 4.**
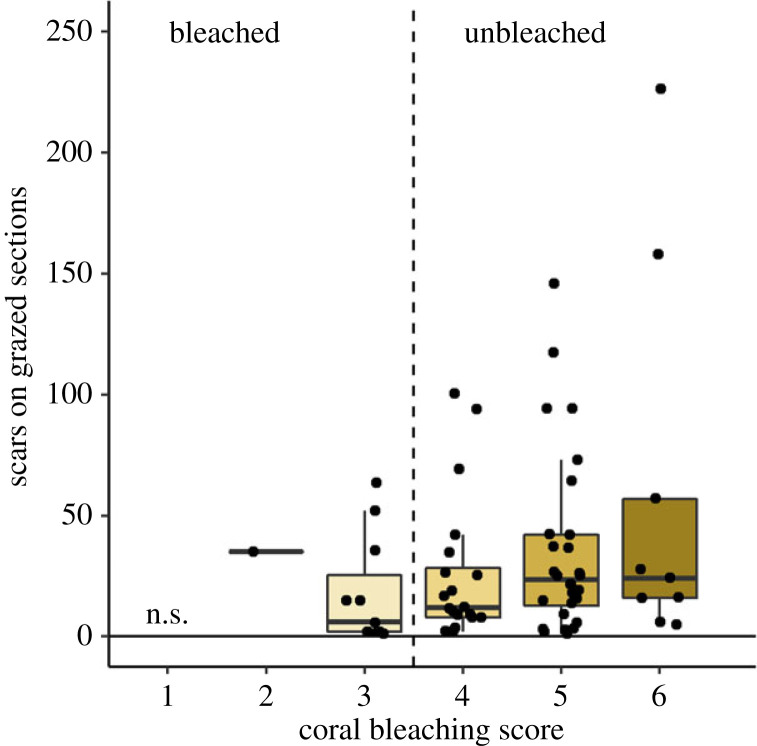
Number of bite scars on grazed coral sections of varying degrees of bleaching in 2019. Each point represents a single coral section. There were no statistical differences in number of scars between sections following least square means tests (electronic supplementary material, table S1). Each point represents the number of bite marks on a single section. Coral sections with a score to the left of the dashed line were considered ‘bleached’ while those to the right were ‘unbleached’. (Online version in colour.)

We recorded no grazing scars on areas of *Orbicella* spp. colonies with a bleaching score of one (most bleached), and we did not observe a significant influence of bleaching severity on grazing avoidance (*p* ≥ 0.175 for all pairwise comparisons; electronic supplementary material, table S1) with parrotfish avoiding bleached *Orbicella* spp. colonies regardless of bleaching score (figure 4). However, there was a trend towards increased grazing on corals with higher pigmentation—the highest number of grazing scars (226 bites) were observed on a single colony with a bleaching score of 6 (not bleached).

### (d) Bleaching severity is not related to coral size

**Figure 5.**
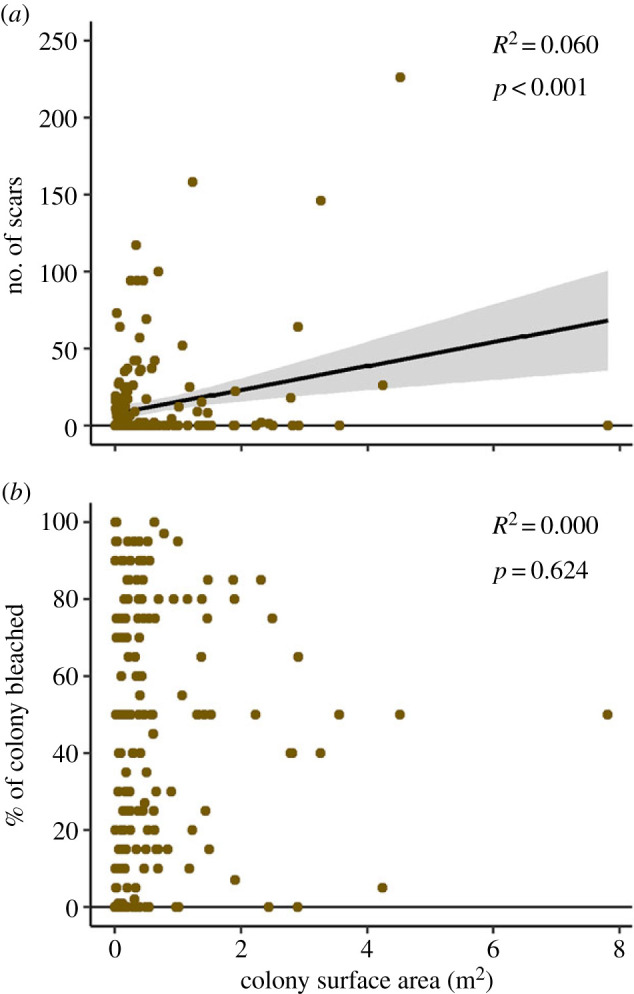
(*a*) Relationship between *Orbicella* colony surface area and the number of scars observed and (*b*) the amount of bleached area on the colony. *R*^2^ and *p*-values calculated using linear regressions. The shaded area around the regression line indicates the 95% confidence interval. (Online version in colour.)

We captured a broad distribution of *Orbicella* colony size—colony surface area ranged from 0.002 to 7.806 m^2^. While there was a significant relationship between coral surface area and the number of grazing scars (*p* < 0.001), this relationship described only a very small amount of the variance (*R*^2^ = 0.060; figure 5*a*). There was no relationship between colony surface area and the per cent of the colony that was bleached (*p* = 0.624; figure 5*b*).

### (e) Heavily grazed colonies may bleach more

**Figure 6.**
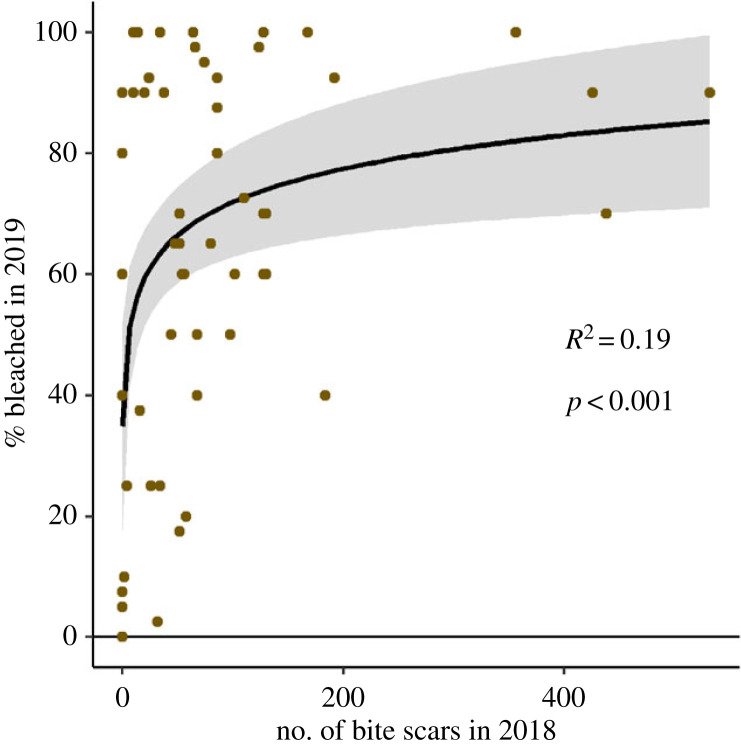
Relationship between the number of parrotfish bite marks on *Orbicella* colonies in 2018 and subsequent bleaching in 2019. *R*^2^ and *p*-values were estimated using a log regression. The shaded area around the best fit line indicates the 95% confidence interval. (Online version in colour.)

A log regression demonstrated that *Orbicella* spp. colonies with parrotfish grazing scars in 2018 had a significant positive relationship (*p* < 0.001; *R*^2^ = 0.192; figure 6) with the area of the same colony that was bleached in 2019.

### (f) Slight difference in nutrient status between bleached and unbleached corals

We found no differences in the relative nutritional status—%C (*p* = 0.904) or %N (*p* = 0.885)—of bleached and unbleached corals (table 2). There was no difference in δ^13^C content of bleached and unbleached corals (*p* = 0.471; table 2), but δ^15^N of bleached corals was significantly enriched (2.45 ± 0.16‰) relative to unbleached colonies (1.56 ± 0.21‰; *p* = 0.006).

**Table 2.** Carbon and nitrogen content and isotopic ratio of unbleached and bleached coral tissue. Values reported as mean ± s.e. For one unbleached sample a C peak was not recorded during sample analysis.

	tissue %C	tissue %N	δ^13^C (‰)	δ^15^N (‰)
unbleached	36.20 ± 3.88 (*n* = 8)	4.97 ± 1.12 (*n* = 9)	–12.42 ± 0.66 (*n* = 8)	1.56 ± 0.21 (*n* = 9)
bleached	36.76 ± 3.69 (*n* = 6)	4.61 ± 0.65 (*n* = 6)	–13.33 ± 1.09 (*n* = 6)	2.45 ± 0.16 (*n* = 6)

## 4. Discussion

Large scale coral bleaching events are causing rapid decline of reefs globally [43,44]. Since the onset of large scale coral bleaching events in 1998 that killed 8% of reefs worldwide, an additional 14% have been destroyed between 2009 and 2018 [45]. It is well understood that coral bleaching is most common on reefs experiencing high intensity thermal stress anomalies [46], which are predicted to worsen under global change conditions [47]. As such, as bleaching continues to stress reefs both in frequency and intensity, it is important to understand the real-time ecological changes to community-level interactions. Community interactions (e.g. corallivore grazing) with corals during bleaching, for example, may have the ability to exacerbate bleaching intensity or severity (this study), or conversely may help bolster coral resilience [48]. Corallivores interact with corals ecologically, mainly through trophodynamics that can shift behaviourally in space and time [17,18]. Because corals are foundation species and important primary producers, their real-time interaction with corallivores during periods of increased vulnerability via bleaching has the potential to ecologically impact reef communities through cascading events, though these interactions have not been well-examined [17–19]. Because behavioural mediation of ecological dynamics can strongly influence species' responses to rapid environmental changes [49], corallivore behaviour (i.e. whether corallivores increase or decrease their feeding activity in response to bleaching), may subsequently influence coral resilience.

From our study, it is clear that corallivorous parrotfishes avoid consumption of live, bleached coral, suggesting either that the trophic benefits of corallivory are severely diminished or erased during bleaching, or that parrotfish do not recognize bleached corals as a viable food source. Our findings are consistent with the only other study (to our knowledge) to examine real-time behavioural responses of corallivores to environmentally induced bleaching [22], who found that three species of obligately corallivorous butterflyfish also actively avoided grazing on bleached corals. While neither study can distinguish between the mechanisms determining fish foraging behaviour, it is plausible that fish might be visually responsive to changes in Symbiodiniaceae species, or symbiont density, as previous work has demonstrated that grazed colonies have lower symbiont densities [37]. MacDonald *et al*. [22] observed depth-dependent grazing selectivity, which may also be consistent with this idea, as host corals are known to shift their association with symbionts across depth gradients [50,51]. Alternatively, MacDonald *et al*. [22] postulate a density-mediated scenario, where higher or lower abundances of corals in shallow water may influence the grazing shift to deeper waters. As such, while bleaching may trigger a change in trophic grazing response, this grazing response might also influence depth-dependent effects of bleaching. Though not tested here, future work should investigate whether there is a shift in grazing from shallow, bleached corals to deeper, unbleached corals that may—if it occurs—increase the susceptibility of those deeper corals to grazing. It is unclear whether deeper corals are more susceptible to bleaching [52,53], or are buffered from bleaching [54–58] compared to shallow corals, but a better understanding of the mechanisms underpinning trophic shifts in corallivory due to bleaching will help elucidate the extent of behavioural impacts in shallow waters and at depth.

We did not observe a significant influence of bleaching severity on grazing avoidance. Instead, we found that parrotfish avoided bleached *Orbicella* spp. colonies regardless of bleaching score. However, there was a trend towards increased grazing on higher pigmentation, consistent with butterflyfish feeding on *Acropora* and *Pocillopora* spp. in Moorea, French Polynesia [22]. A study of 38 butterflyfish species feeding on *Acropora* corals across the central Indo-Pacific found that suppressed grazing was sustained for up to 12-months post-bleaching, with *Acropora* colonies from bleached reefs experiencing up to 85% fewer bites [59]. This reduction in grazing both during ([19]; this study) and after bleaching [59] may signal diminished nutritional benefit to fishes from coral consumption and may necessitate trophic shifts in these species. Further, these diminished colony nutritional resources may also play a role in coral resilience, specifically post-bleaching recovery. Indeed, a previous study found that *Orbicella* spp. corals grazed by parrotfishes recovered more slowly from bleaching compared to their ungrazed neighbours [37]. Although longer-term consequences of grazing and bleaching interactions have not been extensively examined, reductions in coral tissue nutritional quality and nematocyst densities have been reported to persist even eight-months post-grazing [60], suggesting that corals require time—of the order of months—to substantially recover from damage (whether bleaching or grazing). However, in our study, we did not observe any significant differences in overall nutritional quality in real time (although it should be noted that we only measured nutritional quality in regard to %C and %N, which were no different between bleached and unbleached colony sections. Measuring other compounds such as lipids and proteins can provide a more thorough description and quantification of nutritional quality). We did find that bleached colonies had significantly higher δ^15^N than unbleached colonies, suggesting a greater importance of heterotrophy relative to N-fixation as a method of obtaining N by bleached corals [42,61], as heat stress increases energy demands of the coral host [62], and higher overall energy reserves are thought to buffer against environmental stress [28,63].

**Figure 7.**
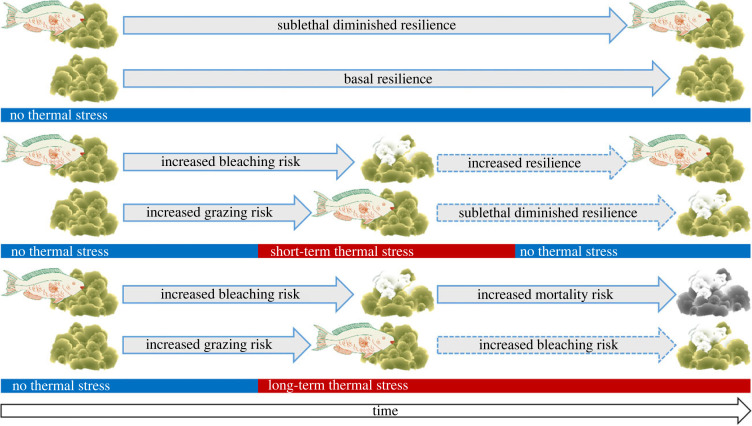
Hypothetical changes to coral bleaching risk and mortality mediated by grazing behaviour under three thermal stress scenarios over time. Top: normal conditions (no thermal stress), middle: short-term thermal stress, bottom: long-term thermal stress. Solid arrows denote observations made in the literature or in this study. Dashed arrows denote hypothetical outcomes. These scenarios express our emerging hypothesis that increased grazing pressure on unbleached corals may elevate coral vulnerability to bleaching (decrease resilience) under scenarios of sustained thermal stress, but the temporary cessation of corallivory on bleached corals may increase resilience in the short-term. Coral and parrotfish symbols are from the Integration and Application Network, University of Maryland Center for Environmental Science (www.ian.umces.edu/symbols). (Online version in colour.)

From the perspective of coral health and resilience, it would seem beneficial that corallivorous grazing is temporarily suspended during bleaching. Suppression of grazing may prevent corals from falling further into debt on their energy budget (energetically balancing wound healing stress with bleaching stress simultaneously). Especially during bleaching, corals are carbon-limited due to dysbiosis [64,65], and allocating additional energy to wound healing might accelerate coral mortality. Previous studies on coral wounds have demonstrated intensive resources mobilized in immune response and wound healing [66–69]. Thus, we hypothesize that temporary grazing suppression during bleaching might increase resilience, but would that resilience be enough to bolster corals towards recovery versus mortality? The answer to this question likely relies, at least in part, on prior coral condition (pre-bleaching; figure 7). Here, we found that *Orbicella* spp. colonies that were heavily grazed in 2018 exhibited greater surface area of bleaching in 2019 compared to intact (ungrazed) colony neighbours, which is consistent with previous evidence that snail corallivory can increase bleaching severity [70]. This intensified bleaching response due to grazing, coupled with the role of grazing in delaying recovery post-bleaching [37], suggests that the temporary cessation of corallivory might not be sufficient to fully spare corals from mortality. Here, we found that the relationship between grazing intensity and bleaching severity described 20% of the variance in bleaching severity (*R*^2^ = 0.19; figure 6), leading to a second hypothesis: that increased grazing pressure on unbleached corals may elevate coral vulnerability to bleaching (decrease resilience) under scenarios of sustained thermal stress (figure 7). Sum total, however, the temporary cessation of corallivory on previously grazed (and thus likely to be more heavily bleached) corals may be enough to tip the balance towards a higher likelihood of post-bleaching recovery than if grazing continued through the bleaching process (figure 7). Although the longer term hypotheses remain to be comprehensively tested, they raise the idea that grazing events—even if grazing occurs pre-bleaching—can have longer-term resilience consequences that may not be fully balanced by the suspension of short-term corallivory.

By contrast to previous findings, [71–73] we did not observe any influence of coral colony size on bleaching severity. There was a significant relationship between coral surface area and the number of grazing scars, and a significant relationship between grazing scars and bleaching susceptibility, suggesting that coral size may indirectly influence bleaching mediated by grazing intensity. This finding suggests that coral morphology alone is insufficient to explain bleaching susceptibility on *Orbicella* spp. corals, which is consistent with previous studies that have documented similarly sized neighbouring *Orbicella* spp. colonies with differential bleaching plasticity and physiology [37,63]. Taken together, corallivore behaviour may play a larger role in bleaching susceptibility than has previously been appreciated.

As climate change continues to warm the planet and destabilize coral reef conditions, it is important to understand how the real-time ecological dynamics of coral bleaching influence the behaviour and ecology of associated reef communities and how in turn, those community responses can impact reef resilience. In this study, we have shown that predator behaviour and trophic shifts may contribute to increased vulnerability of *Orbicella* colonies that would otherwise display higher bleaching resistance. For example, we observed heavy grazing on pigmented (unbleached) coral colonies, even though very few of these colonies were available. Taken together with previous studies, we now posit a conceptual framework on how grazing influences bleaching, and vice versa, to impact the outcome of coral resilience over time (figure 7). In particular, a previous study has demonstrated that parrotfish tend to repeatedly graze the same colonies of *Orbicella* spp. over time [60], and our study shows that previously grazed colonies in 2018 were bleached (and therefore ungrazed in 2019), suggesting a behavioural shift by corallivores to available colonies that would not typically be grazed. This shift in corallivore behaviour might, therefore, mediate coral resilience/vulnerability if there is increased grazing pressure on unbleached colonies, but future studies will have to test this emerging hypothesis, and whether it will differ in the short- or long-term.

## 5. Conclusion

The oceans are continuing to experience more frequent and severe climate stress, and predicting ecosystem responses to these stressors requires a nuanced understanding of changes in species interactions, in part driven by behavioural and trophic plasticity pairwise interactions. Despite the vast literature on coral bleaching and subsequent consequences to reef ecology, little is known about the interaction between bleaching and corallivory and the resulting real-time ecological impacts on trophic transfer from corals to corallivores. This study is among the first to document real-time behavioural shifts of feeding behaviour during bleaching in the wild, and to relate those behavioural changes to downstream resilience or recovery potential. Emerging hypotheses remain to be tested, but reliable predictions of coral changes in response to bleaching should incorporate the trophodynamic plasticity of corallivores as part of the complexity of reef ecosystems.

## Data Availability

The data generated in this study and used in our analyses are stored in the Figshare repository and can be accessed by the following link: https://doi.org/10.6084/m9.figshare.20371677 [74]. R code used in statistical analysis can be accessed via github via the following link: https://github.com/nray17/bleaching_corallivory. Supplementary material is available online [75].
